# The effect of sex and age on facial shape directional asymmetry in adults: A 3D landmarks-based method study

**DOI:** 10.1371/journal.pone.0288702

**Published:** 2023-08-03

**Authors:** Katarína Harnádková, Karolina Kočandrlová, Lenka Kožejová Jaklová, Ján Dupej, Jana Velemínská

**Affiliations:** 1 Laboratory of 3D Imaging and Analytical Methods, Department of Anthropology and Human Genetics, Faculty of Science, Charles University, Prague, Czech Republic; 2 Department of Histology and Embryology, Third Faculty of Medicine, Charles University, Prague, Czech Republic; Universiti Sains Malaysia, MALAYSIA

## Abstract

**Objectives:**

Facial directional asymmetry research, including age-related changes, is crucial for the evaluation of treatment of craniofacial malformations/trauma in orthodontics, facial surgery and forensic sciences. The aim was to describe facial directional asymmetry (DA) in different age categories of adults using 3D methods. According to our hypothesis, facial **shape DA** (1) depends on sex; (2) differs among age groups; and (3) has wider variability in older age.

**Material and methods:**

A cross-sectional sample of healthy Czech adults without craniofacial trauma or anomalies consisted of 300 3D facial models (151 females). The age-range in the study was between 20–80 years. The shape asymmetry of 28 3D landmarks was evaluated using geometric morphometrics and multivariate statistics.

**Results:**

The manifestation of DA was similar in both sexes and in each age category; however, there were some statistical differences. In contrast to the ideal symmetrical face, the mean asymmetrical faces tended to create a slightly bent “C” shape of the midline. Therefore, the upper face was rotated slightly clockwise and the lower face counter-clockwise. The right eye was located slightly higher, with the nasal tip and mandibular region tilting to the left. Sex differences in facial DA were significant before the age of 40. DA was more significant in the youngest males than in the oldest, while the women’s DA did not change.

**Conclusions:**

The DA patterns were similar in both sexes and in all age categories (a slightly bent C shape of the midline); however, some significant local differences between male age groups were found. A significantly more pronounced asymmetry compared to other age groups was found only in the youngest males from 20 to 40 years. Moreover, significant sexual dimorphism of DA rapidly decreased after middle age, likely caused by the same age-related changes of the face during aging.

## Introduction

A detailed description and deeper knowledge connected to the human face can provide information that is necessary in medicine [1, 2], a forensic contexts, and biometry [3]. Facial asymmetry also has the potential to describe significant partial details of the facial area needed by many specialists in orthodontics [4], as well as reconstructive and plastic surgery [5]. Moreover, DA can significantly affect craniofacial complex and even in healthy individuals there is a certain level of directional asymmetry. DA could significantly affect aesthetics or function of the facial region and facial surgeons are focusing on it to achieve the best possible results of the surgery [5]. With desire for facial surgery (and facial asymmetry correction) is related fact that humans are very sensitive to perceive facial asymmetry. Perceptive thresholds are different for facial areas, where eyelid position asymmetry is perceived (the most sensitive) at 2 mm and chin deviation is perceived at 6 mm [6].

Despite the presence of facial asymmetry (normal asymmetry) in each individual [7], the higher levels of asymmetries (pathological) are often associated with dentofacial deformities and may cause many functional problems of the facial area [8]. Dentofacial deformities or disabilities associated with craniofacial asymmetry include unilateral condylar hyperplasia [9], skeletal classes II and III [8], hemifacial microsomia [10], cleft lip and palate [11] and facial palsy [12]. Appropriate individual treatment leading to more symmetrical faces is fundamental in order to increase the functionality of the craniofacial complex, and the feeling of comfort and attractiveness in patients [7]. During facial surgery planning, it is essential to recognize and determine the facial asymmetry of the patients because the aim of surgeons is to provide as realistic and aesthetic treatment outcome as possible. The precise description of the facial asymmetries could help with predictions, even if the whole facial asymmetry is not an object of interest. One of the most common surgeries in all age categories is rhinoplasty, in which only one area is operated on [5, 13]. Facial standards/references are useful for comparing possible facial disproportions (in both sexes and at different age stages) with normal faces, providing an insight into the healthy population. Standards are also useful for comparisons of diagnosis or disproportion levels [14, 15]. Very significant groups where the facial asymmetry is a relevant factor are lip and palate cleft disabilities. In this area, it is absolutely essential to know how overall facial directional asymmetry and the asymmetry of affected areas occur and develop during aging, or how much they differ from healthy individuals [16].

Moreover, based on facial asymmetry and other traits, variables like attractiveness, fitness [5] and age [17, 18] can be described or predicted. Studies dealing with facial aging usually focus on overall morphology. However, the association of aging and facial asymmetry is not common in the literature despite the possibility of using this knowledge in patient treatment.

Facial asymmetries occur and change throughout growth and aging. They are a reflection of lifestyle [19], congenital traits and developmental events [20]. Facial asymmetry is described as an age-dependent trait increasing with age [1, 18]. The age-related changes in children and adolescents are strongly affected by craniofacial growth, maturation, and dental eruption. In adults, age-related facial changes are mostly linked to degenerative processes [3]. Studies suggest that sexual dimorphism in facial DA occurs despite the fact that the differences are not great. Usually males show wider variability of asymmetry than females [21, 22]. However, there is also a study where sexual dimorphism in facial asymmetry is not proven [23].

Significant morphological changes of the craniofacial complex occur in adults after 60 [3]. These changes include a decline of the bone mass from mastication, gravity [17], and dentoalveolar changes [24]. Bone changes occur primarily in the forehead area, with wider convexity in females. The supraorbital area and glabella are more protrusive in males [25]; there is also orbit enlargement, upper jaw reduction [17], and nasal area change [24]. For soft-tissue between 30 and 60, the most intensive deterioration of tissue volume is in the infraorbital, lateral, medial cheeks, and temporal area [26]. Between 40 and 50, the eyebrows and upper eyelids drop. Moreover, the skin quality [24] and lip thickness decline with increasing age [27]. The lower jaw is most affected by gravity, which is the most crucial source of facial asymmetry with vertical movements in the upper lip and gonions [28].

The asymmetries of the facial hard and soft tissue are closely linked, with both usually occurring [29]. Facial shape asymmetry tends to be most dominant in the lower jaw region, less in the middle face region, and least in the upper region [1, 8, 18]. The chin tends to deviate to the left [30], although the tragus and gonions tend to the right side [31]. The nose mainly tends to the left side [5, 32], with the left facial side often showing more asymmetrical features. This could be explained as a correlation between asymmetries of the sides of the brain, where emotions are controlled in the right hemisphere, thus the functional manifestation of this being observed on the opposite (left) side [5, 33]. Similar cross-asymmetry has been described in speech (located in the left hemisphere) and mouth opening, which is more frequent on the right side [34].

There are two perspectives on shape asymmetry assessment. The first is matching symmetry, where two separate copies from the left and right sides exist, while the symmetrical axis passes between them [35, 36]–this could be patterned for the human hand. The second perspective is object symmetry, which is specific for the human face [37]. This kind of a/symmetrical pattern represents an object that is symmetrical in itself (having two mirrored halves), with a symmetrical axis running through the whole object [35, 36]. Landmark configurations consist of paired (the same landmarked structures on the left and right sides) and median ones. Before the Procrustes fit, the original landmark configuration is reflected and relabelled into a mirrored landmark configuration; then the original reflected and relabelled configuration creates an ideally symmetric configuration (consensus shape) together. This allows us to compare the new mirrored configuration with the original shape, or to compare an original configuration with an ideally symmetrical configuration. This comparison and quantification of differences represents the asymmetrical component–shape asymmetry [36].

This paper presents a 3D-landmark-based approach for assessing sex and age-related changes in facial shape DA, in which we describe adult cross-sectional data (20–80 years of age). The paper calls into question how facial asymmetry changes at various adult ages and whether there are some sex differences throughout adulthood. Our first hypothesis was that facial DA depends on sex. The second hypothesis was that facial DA differs among age groups. An understanding of facial asymmetry at various ages is fundamental in orthodontics, facial surgery planning, aesthetics treatment and in the identification of individuals. Every period of an individual’s life is different with specific facial changes; with more precise description of age-related changes, it will be much more effective to plan facial treatments. Moreover, 3D methods allow us to evaluate the face much more realistically than 2D methods.

## Materials and methods

### Ethics statement

The subjects participated voluntarily and gave signed written consent for the study (with agreement to long-term research). The study was given written approval by The Institutional Review Board of Charles University, Faculty of Science, Prague, Czech Republic.

### Subjects

Exactly 300 3D facial surface scans of Czech adults (collected from 2009 to 2015) were analysed. The cross-sectional sample (151 females, 149 males) selected from our laboratory database of 3D facial scans consisted of healthy, European adults born in the Czech Republic without craniofacial or dental anomalies, traumas with consequences, facial/dental surgery, or dermal fillers. Subjects with significant changes in tooth number or other pathological facial conditions were excluded from the sample. Subjects’ ages ranged from 20 to 80 years and were divided into three age categories (**Table 1**). The BMI (body mass index) of the adults was between 18.5 and 25. According to World Health Organization (WHO) classification, this BMI range is considered a normal weight [38].

**Table 1:** Age distribution of the sample with its statistical indicators. N–number of subjects; SD–standard deviation. Age groups: A (20–39.99 y), B (40–59.99 y), C (60–79.99 y). SD = standard deviation.

Age category	Sample size	Age range	Median	Mean	SD
**MALES N = 149**					
**A**	91	20–39.99	23.80	25.66	5.14
**B**	35	40–59.99	49.50	49.51	5.33
**C**	23	60–79.99	66.75	69.79	7.42
**FEMALES N = 151**					
**A**	56	20–39.99	24.40	26.03	5.54
**B**	47	40–59.99	48.90	48.63	5.57
**C**	48	60–79.99	70.05	70.42	6.99

### Data acquisition

Subjects without facial hair were scanned by the 3dMDface System optical scanner with a synchronised dual-camera system. The scanner can automatically create a continuous polygonal 3D mesh of the face with appropriate colour texture. Technical parameters of the 3D scanner were following. 3D model geometry accuracy was established at <0.2 mm or better. Field of view was ear-to-ear, 180-degree face capture. The 3D mesh was with x, y, z coordinate system from all synchronized stereo pairs–six machine vision cameras and industrial-grade flash system, which are synchronized in a single capture. One capture takes a speed ~ 1.5 milliseconds and final 3D Image rendering speed was 7 seconds. Quality of 3D meshes can be affected by subject movement during the duration of the scanning or by facial hair–can cause errors and holes in meshes. These limitations were eliminated by individual rescanning if there was any movement during scanning and by exclusion of individuals with facial hair.

**Fig 1:**
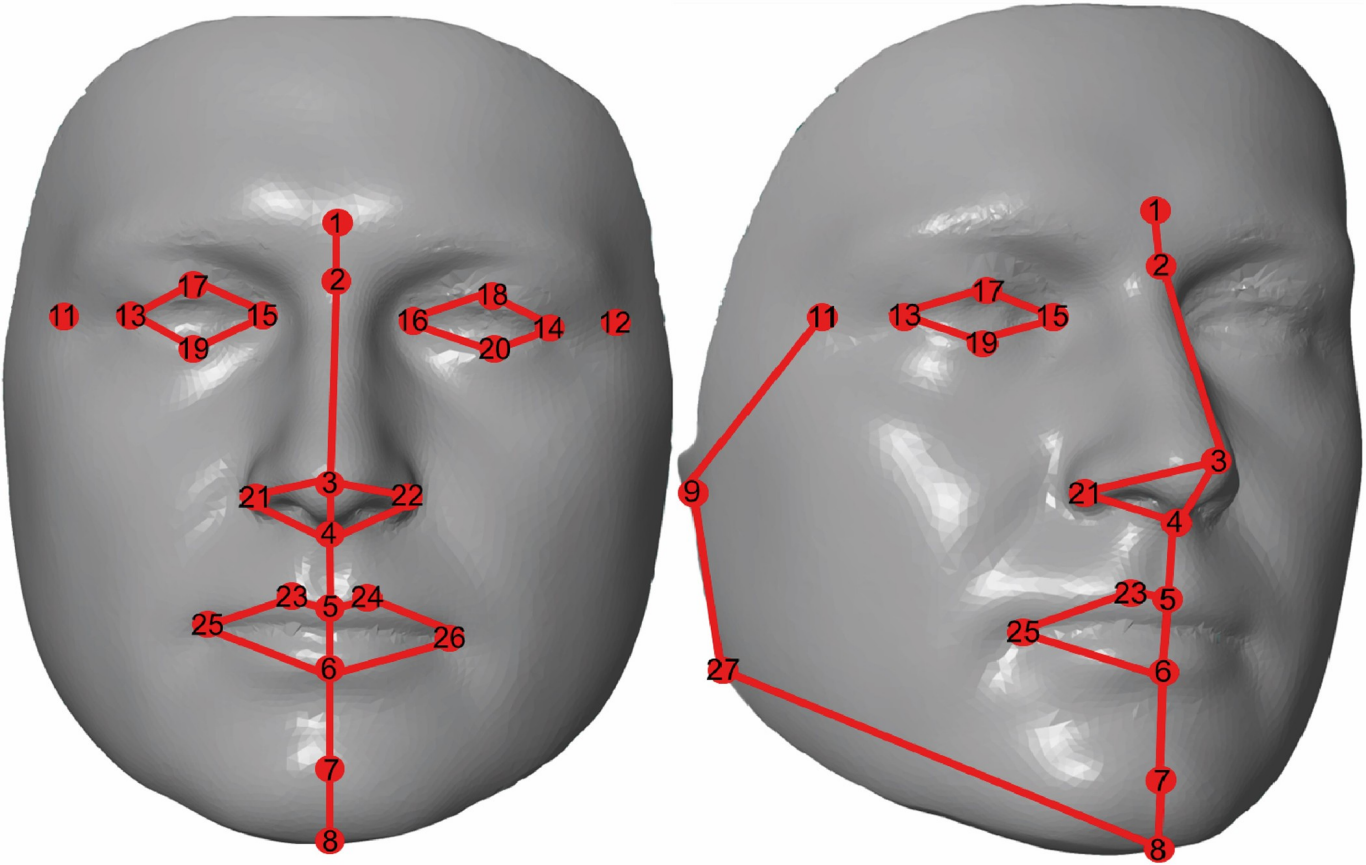
Shows the localization of the 28 used 3D landmarks.

Optical scanning is a safe non-invasive method for living subjects. They were scanned while seated, from a frontal view, with a neutral facial expression (mouth closed) by one technical operator. Post-processing was done according to Velemínská’s methodology [39]. The morphology of the faces was described by anatomical landmarks only–they are more reliable for digitalization and reproducibility. A total of 28 3D landmarks were placed on the facial polygonal surface (**Table 2 and Fig 1**). The Cartesian coordinates (x, y, z) of all 28 landmarks were digitized by the same technical operator. For intra-observer measurement error (landmark placement error), landmarks were digitized five times on 9 3D models (i.e. on 3 models from age group A, 3 from age group B, and 3 from age group C) by two different technical operators. The first technician digitized the whole dataset. The inter-observer landmark placement error was performed on the same sub-dataset as the intra-observer landmark placement error by the same two technician operators. Morphome3cs software was used to process the measurement error in millimetres according to von Cramon-Taubadel et al. (2007) methodology [40].

**Table 2:** A list of the 28 3D landmarks. Landmarks 1–8 were unpaired. Landmarks 9–28 were paired–the same landmark was digitized on the right and left sides.

Name	Abbreviation	Number	Definition
**UNPAIRED LANDMARKS**			
**Glabella**	g	1	The most prominent mid-point between eyebrows
**Nasion**	n	2	The innermost landmark between nose and forehead
**Pronasale**	pr	3	The most prominent point on the nasal tip
**Subnasale**	sn	4	The mid-point of the columella base
**Labrale superius**	ls	5	The mid-point of the upper vermilion border
**Labrale inferius**	li	6	The mid-point of the lower vermilion border
**Pogonion**	pog	7	The most prominent median point of the anterior side of the chin
**Gnathion**	gn	8	The most inferior mid-point on the lower border of the chin
**PAIRED LANDMARKS–for right and left sides**			
**Otobasion inferius**	obi	9 and 10	The most inferior point of the attachment of the cheek with the ear lobe
**Frontozygomaticus**	fz	11 and 12	The innermost point on the frontozygomatic suture area
**Exocanthion**	ex	13 and 14	The most lateral corner of the eye fissure
**Endocanthion**	en	15 and 16	The most medial corner of the eye fissure
**Palpebrale superius**	ps	17 and 18	The highest point on the free margin of the upper eyelid
**Palpebrale inferius**	pi	19 and 20	The lowest point on the free margin of the lower eyelid
**Alare**	al	21 and 22	The most lateral point on the ala of the nose
**Crista philtri**	cph	23 and 24	The point on the elevated margin of the philtrum above the vermilion border
**Cheilion**	ch	25 and 26	The most lateral point at the angle of the vermilion border
**Gonion**	go	27 and 28	The lowest point of the lower jaw at the mandibular angle

Using MorphoJ software [41], the Cartesian coordinates were aligned by Procrustes fit (Generalised Procrustes Analysis) and the statistical analysis was performed here–Procrustes ANOVA and Principal Component Analysis. Statistics such as Hotelling’s T^2^ test (T-squared distribution), MANOVA (Multivariate Analysis of Variance), the Henze-Zirkler test, and Box’s M test were calculated in R software [42]. For each analysis the whole landmark configuration (all 28 landmarks) was used. In the Procrustes ANOVA, the asymmetrical component from landmarks was used and the rest of the analyses used PCA score from PCA analysis (calculated from the whole landmark configuration as well).

The landmark digitization was carried out in GOMInspect software [43], with the statistical significance level set at 5% for most of the tests, the exception being the statistical significance level set at 0.1% for Box’s M test. Bonferroni correction for multiple comparisons [44] was set at 0.0083 for multiple Procrustes ANOVA (significance level 0.05/number of tests 6 = significance level after correction 0.0083). Another Bonferroni correction for multiple comparisons was set at 0.01667 for multiple Hotelling’s T^2^ tests (significance level 0.05/number of tests 3 = significance level after correction 0.01667). Assumptions for parametric statistical testing were verified by the Henze-Zirkler test (normality) and Box’s M test (equivalence of covariance matrices).

### Geometric morphometrics and statistical analyses

The Cartesian coordinates were aligned by the Generalised Procrustes Analysis (GPA), and the object a/symmetry shape model was used with two sets of landmarks in configuration: 1) unpaired landmarks and 2) paired landmarks (for both sides of the face). After the GPA, the Cartesian coordinates were divided into symmetrical and asymmetrical shape components–used in the study analyses. This shape component represents the difference between the ideally symmetrical (symmetrical component) and the original landmark configuration. Sexes were assessed separately in analyses. The first step of the statistics was Procrustes ANOVA (Procrustes analysis of variance); it assessed the presence/absence and manifestation of DA in tested groups separately: Males A group, males B group, Males C group, females A group, females B group and females C group– 6 different Procrustes ANOVAs. In each analysis were used only the default variables, while the most important were individual effect and DA effect. Principal component analysis (PCA) of the asymmetrical component was performed for a reduction of data dimension; principal components (PCs) were used for further statistical methods. In subsequent analyses, PCs were included to provide cumulative variability for at least 80% (the first 10 PCs) of overall sample variability.

The differences in average shape asymmetry between groups (sexes, between age groups–A, B and C), represented by a Principal Component Score of the first 10 PCs, were statistically tested by Hotelling’s T^2^ tests and MANOVA (with post-hoc tests–Hotelling’s T^2^ tests).

## Results

### Normality tests and landmark placement error

Assumptions for parametric testing were not violated; Box’s M test (A age group p value = 0.1194; B age group p value = 0.4664; C age group p value = 0.0824; p value for males = 0.0510 and p value for females = 0.0013); the Henze-Zirkler test (A age group p value = 0.6002; B age group p value = 0.0849; C age group p value = 0.0735; p value for males = 0.7438 and p value for females = 0.2239).

The mean landmark placement error for the first author of the paper, who digitized the data set for asymmetry analysis, was determined at ± 0.3320 mm. The mean landmark placement error for the second technical operator was determined at ± 0.5781 mm. The mean inter-observer error between these two observers was determined at ± 0.6817 mm [40]. Neither the intra-observer- nor the inter-observer landmark placement error exceeded 1 mm.

### Manifestation of shape directional asymmetry

**Fig 2:**
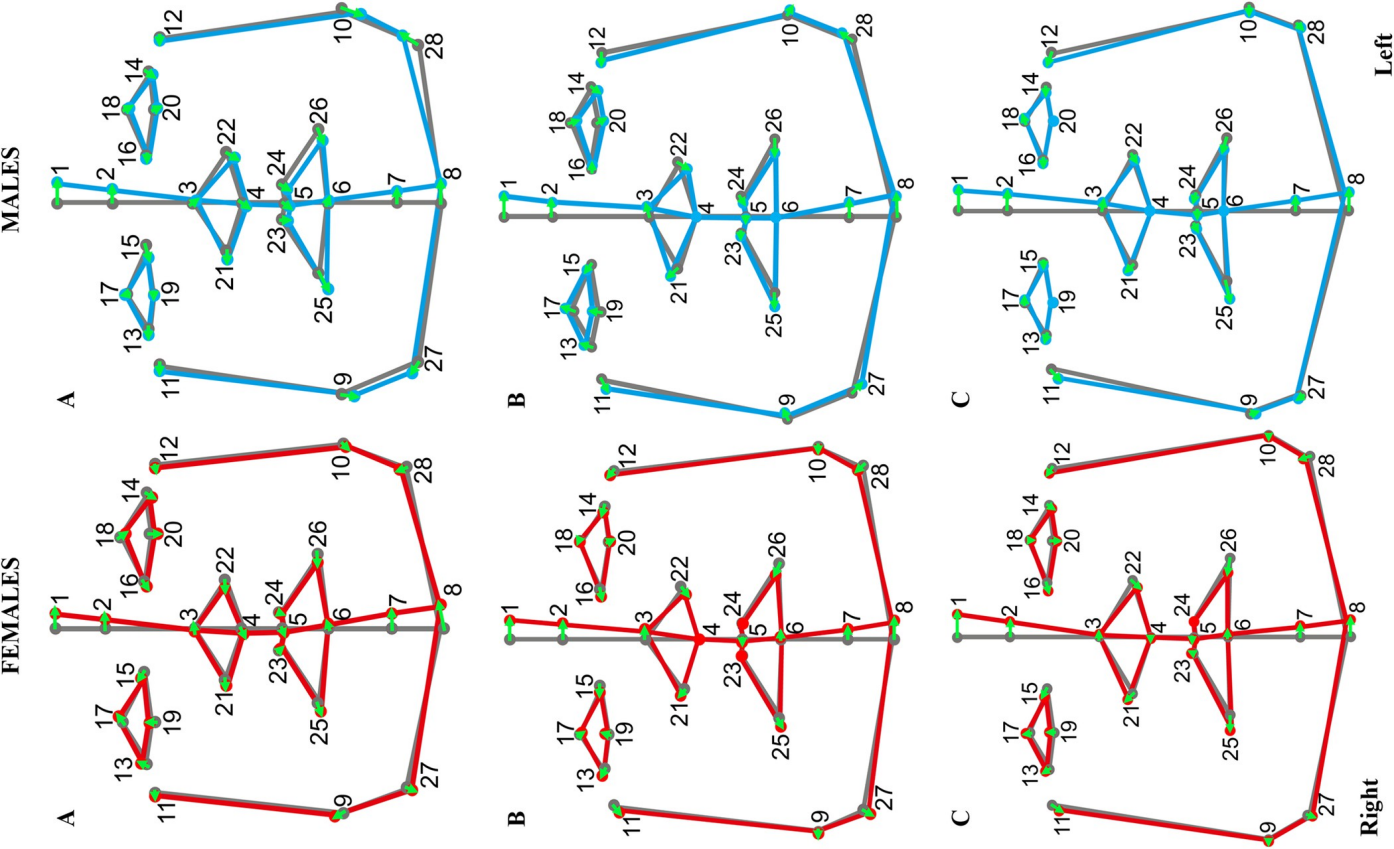
Mean facial asymmetry from the frontal view for males (blue wireframe graph) and females (red wireframe graph). The grey wireframe graph shows the mean symmetrical shape for the whole group. The blue and red wireframe graphs show the specific directional asymmetry in shape beside the group-specific symmetrical shape. The green arrows show the direction and range of the asymmetrical shifts from the mean facial symmetry. Age groups: **A** (20–39.99 y), **B** (40–59.99 y), and **C** (60–79.99 y). Numbers 1 to 28 represent 3D landmark numbers. The scale factor for mean facial asymmetry was set at 5 (the default scale factor was 1).

Procrustes ANOVA showed statistically significant DA for the sexes and in each age category separately (p value < .0001 for each tested group; **Table 3.**), which means that DA was present in both sexes and in each group. Average a/symmetrical faces from the frontal view for both sexes in each category are shown in **Fig 2**. These faces represent the mean pattern of asymmetry in each tested group. The general pattern of the mean DA was similar for each age category and for both sexes, but with some statistical differences among groups. The DA in the frontal view in each group was manifested into a very open C shape. In the upper part of the midline, the glabella tended to the left, with the nasion and pronasale less so. The middle part of the midline (subnasale–labrale inferius) returned to the symmetrical mean; however, the lower part of the midline also tended to the left side. Sets of landmarks represented the upper part of the face–the eyes and nose were rotated slightly clockwise. Landmarks on the left side were located higher than on the right side, especially the exocanthions and alares. On the other hand, the landmark sets of the lower face–mouth, jaw and chin–were rotated counter-clockwise. The chin (gnathion) was located the most to the left.

**Table 3:** The asymmetry results from 6 Procrustes ANOVAs for sexes and age categories separately with effects of individual variable (individual effect), and directional asymmetry (DA effect). SS = Procrustes sum of squares; MS = Procrustes mean squares; df = degrees of freedom; F = Goodall´s F statistic; p = p value.

Effect	SS	MS	df	F	p
**Males A**					
Individual	0.5044	0.0001	3822	8.88	**< .0001**
DA	0.0058	0.0002	35	11.32	**< .0001**
**Males B**					
Individual	0.1806	0.0001	1428	5.57	**< .0001**
DA	0.0060	0.0002	35	7.58	**< .0001**
**Males C**					
Individual	0.1330	0.0001	1092	5.45	**< .0001**
DA	0.0044	0.0001	35	5.68	**< .0001**
**FEMALES**					
**Females A**					
Individual	0.2309	0.0000	2394	4.66	**< .0001**
DA	0.0088	0.0003	35	12.12	**< .0001**
**Females B**					
Individual	0.2449	0.0001	2100	2.85	**< .0001**
DA	0.0063	0.0002	35	4.36	**< .0001**
**Females C**					
Individual	0.2834	0.0001	2520	5.01	**< .0001**
DA	0.0084	0.0002	35	10.63	**< .0001**

Based on **Fig 2**, we observed the manifestation of the DA in groups A and B, with slightly more asymmetrical eye, nose and mouth areas, whereas in group C, these areas were less asymmetrical (for the sexes separately). In group C, the DA was quite stabilised, but the face midline was still C-shaped. The eyes, mouth and nose were much closer to the symmetrical mean in age group C than those in A or B. Moreover, the differences in DA manifestation between the sexes were smallest in age group C. Based on the cranio-caudal view of directional asymmetry shape means, it was observed that the left eye was more in front (beside the symmetrical face) than the right eye. The strongest manifestation of the front eye position was in group A, especially in males. In group C, this trend was weak and the DA was close to the mean symmetry shape. The right gonions were more in front than the left gonions (beside the symmetrical face). This trend was most remarkable in the youngest subjects–group A; in males this trend was slightly more significant.

Hotelling’s T^2^ test showed a statistically significant sex difference in DA only for age group A, with p value = 0.0079. In groups B and C, there were no significant sex differences (p value = 0.1743 and p value = 0.1803, respectively).

The results of the MANOVA (for the sexes separately), where differences between age categories were tested, showed statistically significant differences only in males (p value = 0.0020). Post-hoc Hotelling’s T^2^ tests showed that in males there were statistically significant differences in DA between age groups A and B (p value = 0.0078), and between A and C (p value = 0.0076). Between age groups B and C, there was no statistically significant difference (p value = 0.6300). Therefore, facial shape DA was the most similar in older males between age groups B and C.

In females, MANOVA showed no statistically significant differences (p value = 0.8964) among age groups.

### Shape DA variability description

**Fig 3:**
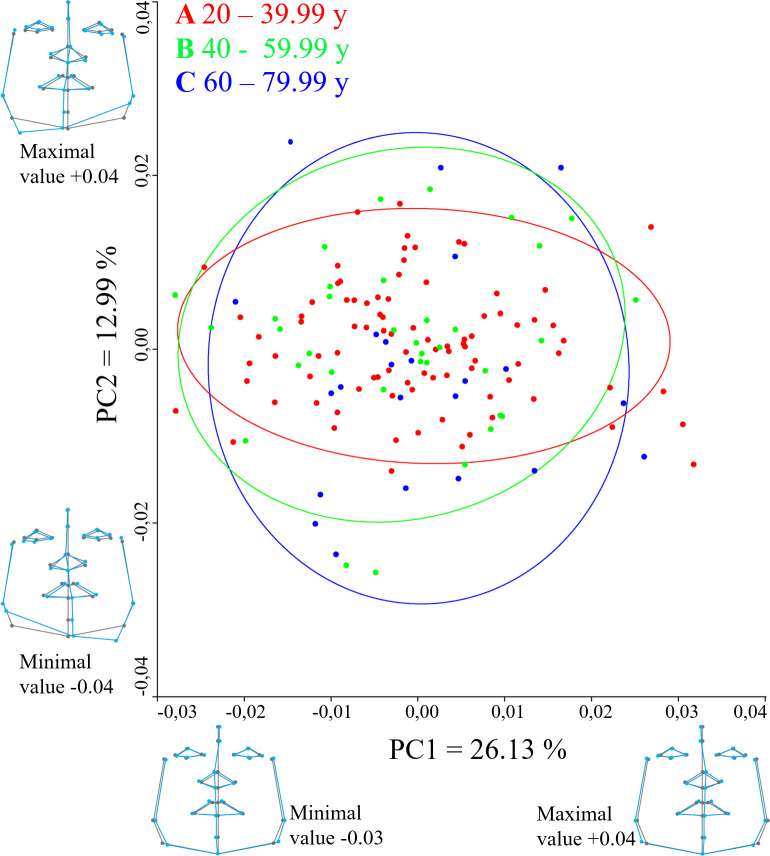
A PCA scatter plot visualizing the variability of shape directional asymmetry in the space of the PC1 and PC2 for males with percentage of variance. The 95% confidence ellipses represent age groups (A, B, C) in years of age. The wireframe graphs show shape asymmetry (blue lines) versus shape symmetrical mean (grey lines) for PC1 (from negative value -0.03 to positive value 0.04) and PC2 (from negative value -0.04 to positive value 0.04).

PC scores divided into age groups (A, B, C) in males are shown in **Fig 3**. Group A, the youngest males, tended to be less variable than males in group C, where facial DA tended to be more variable. The PCA showed that more than 26% of the asymmetrical shape variance was explained by PC 1 (the first PC), and more than 12% of DA shape variance was explained by PC2 (the second PC). The DA shape variance in PC1 was represented primarily by changes in the midline position. The pronasale and subnasale landmarks tended to deviate between the right and left sides the most; a similar trend was observed in the labrale superius and inferius, and pogonion landmarks. The trend of right-left shifting was also visible in the landmarks of jaw contour (otobasion inferius and gonions). PC2 was represented by the deviations in the lower third of the midline (right-left). Significant shape shifts were also evident in the cranio-caudal direction for the eyes, the ala of the nose, and primarily the gonions.

**Fig 4:**
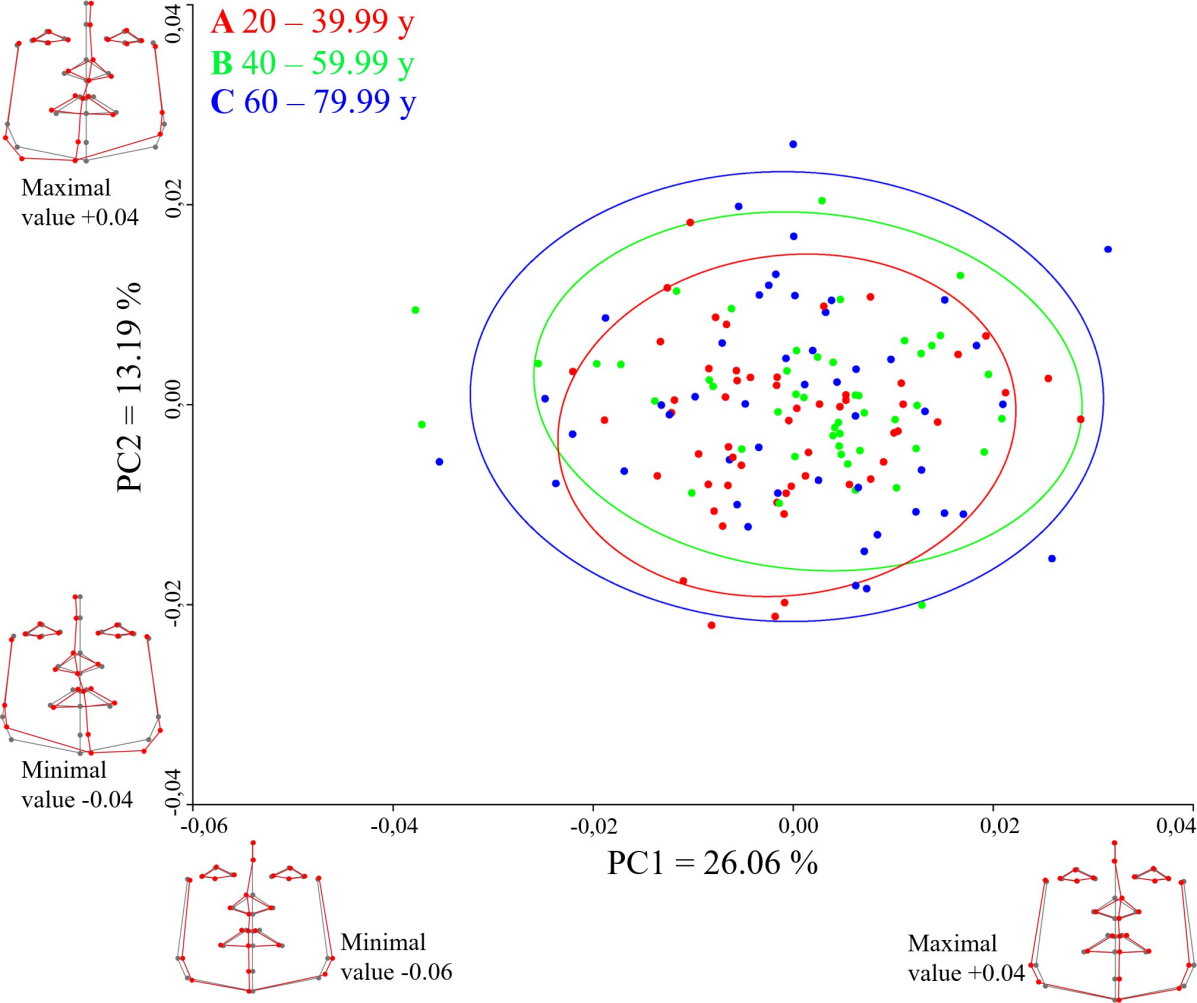
A PCA scatter plot visualizing the variability of shape directional asymmetry in the space of PC1 and PC2 for females with percentage of variance. The 95% confidence ellipses represent age groups (A, B, C) in years of age. The wireframe graphs show shape asymmetry (red lines) versus shape symmetrical mean (grey lines) for PC1 (from negative value -0.06 to positive value 0.04) and PC2 (from negative value -0.04 to positive value 0.04).

The PC scores divided into age groups (A, B, C) in females are shown in **Fig 4**. The youngest females (group A) were less variable than those in group B. Females in group C showed the widest shape variability of DA. The PCA showed that more than 26% of the asymmetrical shape variance of the females was explained by PC1 and more than 13% of DA shape variance was explained by PC2. The DA shape variance in PC1 was primarily represented by changes in the lower jaw contour and in the midline, where the maximal and minimal values of PC reaches side deviation. The most significant side deviation was in the pronasale landmark. The PC 2 was mainly represented by the right-left deviation of the lower facial third and the midline. The upper part of the midline was deviated to one side (right or left–depending on whether there was a negative or positive value of PC). Then in the subnasale landmark, the midline crossed to the other side of the face. Therefore, the lower jaw right-left deviation was contralateral to the right-left deviation of the upper part of the midline. On the deviated side, there was a shift in the caudal direction (e.g., when the lower jaw contour was deviated to the right, it was shifted caudally at the same time).

## Discussion

Articles dealing with age-related changes in facial asymmetry are not common [1, 23] despite the facts that manifestation of facial asymmetry is connected with age-related changes [1, 18] and that the precise evaluation of facial asymmetry is crucial for patient treatment [4, 5, 7]. We aimed to deal with this problem on a 3D level using a geometric morphometric for shape asymmetry evaluation. The modern method utilising 3D facial scans represents a very precise alternative to 2D methods. Many papers have dealt with aging and/or age-related changes presented by morphological [28] and asymmetrical changes [30] employing only 2D images. Scans provide a detailed facial view that allow for the ability to capture every deviation in asymmetry. Additionally, the 3D landmarks method, using almost all of the anatomical landmarks of the face, provide a very quick and well-established approach for the study of asymmetry. It is a method that allows us to describe side and/or antero-posterior deviations; therefore, it has the potential to supplement 3D-mesh methods (there are visible pro/retrusive changes). This detailed description of asymmetry is necessary for facial corrections like the repositioning of the jaws, or surgery planning for the appropriate treatment of pathological patients (cleft lip patients or hemifacial microsomia), or patient treatment after trauma (fractures, dentition loss).

This study is useful in providing us with a quick and current view of the recent population, which is the target population when treating craniofacial malformations [45]; when monitoring genotype-phenotype manifestations of craniofacial deformities [46]; and when planning maxillofacial surgery [47]. The presence of directional asymmetry was found in the whole subject sample, which is consistent with the idea of a lack of symmetry in faces in general [7]. Our results represent population trends (mean facial asymmetries) and show that the overall pattern of facial shape asymmetry was manifested similarly within each tested group (in spite of significant sexual dimorphism and age-related differences in some age categories); however, there were some differences in several areas. In many studies, the lower face is described as the most asymmetrical [1, 18, 31], with the deviation of the lower jaw often being evaluated in orthodontic patients [4]. The accuracy of age estimation increases from the upper to the lower face; this means that the lower part, especially the mandible, is the most asymmetrical facial part [18]. We also described strong asymmetry in the lower facial third, with a trend to side deviations, as presented by Severt et al. (1997) [8]. On average, chin deviation was usually to the left side, which is consistent with the facial asymmetry described in photographs [4, 30] where the left-sided deviation of the chin was also independent of sex and age [4], as in our results. On the other hand, the mean right-side deviation is usually more often evident in the tragion and gonion regions [31]. On the population level, our results manifested ambiguous right-side deviation in all areas except for the centre of the midline, where the landmarks were near to the ideally symmetrical mean. In general, the facial midline showed a pattern similar to the shape of a slightly bent letter “C”. The nose tip tended to the left side in general, which is consistent with other studies [5, 32], although the deviation intensity of the nose varied. The noses in the youngest and the oldest females were only slightly deviated. Overall, the slightly bent “C” asymmetry pattern created a capacity for the larger right half of the face. On the right side of the neurocranium, growth is faster and could manifest into a larger size [48]. Moreover, the right facial side is described as larger, while the left has more asymmetrical features [5]. Apart from the faster right-sided head growth and subsequent increase of final proportions, there is another assumption for facial asymmetry with left-side deviation–mastication [49]. Side preference can vary from side to side [50], but a population trend is noticeable in our sample. Moreover, side preference for the mimic muscles could also affect facial soft-tissue asymmetry [33].

It was noticeable that the upper part of the face and nose were rotated slightly clockwise, and that the lower part of the face was rotated counter-clockwise–in both sexes and in each age category. The rotation of the upper face with the right eye slightly higher than the left one was similarly described on a sample of a medieval skull for which the authors indicated that the upper face could also have been affected by mastication [51]. The lower jaw can generally be affected by pterygo-masseteric sling, which is related to mastication. One historical skull sample also described a similar situation with significant areas affected by mastication with strong right-side preference [52]. Even the post-operative effect on mandible rotation caused by masseter and medial pterygoid muscles was described [53]. Facial asymmetry was suggested as a strongly age-dependent feature that increases with age [1, 18].

Our results showed only partial agreement. Facial asymmetry in our sample was significantly different among some age groups in males, which pointed to age-dependent changes in adults (with regards to most variability included into PC1 and PC2). However, we did not attribute the increase of facial asymmetry to age. In the oldest subjects, our results showed the opposite effect when the midline still manifested into a slightly bent “C” shape, while other regions of the face were closer to the ideally symmetrical mean shape. The whole symmetrical and asymmetrical mean followed the age-related changes of the soft tissue. We observed decline mostly in exocanthions, cheilions, and gonions. These changes were probably related to gravity adjustment and/or quality decline of the skin [24, 27]. However, we are not able to distinguish if the facial changes were related only to physical factors, or whether there were other factors, such as hormonal changes related to aging. Our results showed age-related changes of facial asymmetry only in males, this may suggest that these changes are related to significant morphological changes that are stronger in males than in females. This means that female’s facial asymmetry does not change significantly during adult life, thus processes connected to female morphological aging are not as significant as in males.

Other factors that could affect facial asymmetry are genes or overall genetic background [54–56]. The lower facial third, especially the lower jaw, was assessed as the most asymmetric facial part [1, 8, 18, 28]; in our study we also described the lower jaw as a significant source of asymmetry. Besides the external factors described above, there are gene mutations (ACTN3, PITX2) with at least a moderate degree of impact on mandibular asymmetry [55]. Another study focused on the relationship between facial asymmetry and heterozygosity in an admixed population, which showed that more heterozygous individuals exhibit lower levels of facial asymmetry. Moreover, they suggest some specific patterns of facial asymmetry, which characterize the different ancestry groups [54]. On the other hand, there is a study which does not confirm the assumption that heterozygosity reflects the asymmetries [56].

The results showed significant sex differences, but only in the youngest adult subjects. Moreover, these males, between 20 and 40 years, had slightly more asymmetrical areas (nose, mouth, and lower jaw contours) than females. The faces of both sexes were more symmetrical (especially in the eyes, nose and mouth areas) with age, and sexual dimorphism in facial DA can be dismissed after the age of 40 years. These results are in contrast to the studies [1, 23], where no sex differences are proposed, but differences are found between the sexes in facial asymmetry during the making of expressions [22]. Females did not have as significant changes of facial morphology as males during growth and maturation [57]. This could be reflected in the fewer asymmetrical female faces.

Overall, facial morphology is significantly affected by androgens [58], which could lead to the assumption that facial asymmetry is affected by sex hormones. This also contributes to the conclusion that with the beginning of the decline of sex hormones, after 40 years of age, facial asymmetry becomes more similar between the sexes.

This study was limited by a cross-sectional sample; nevertheless, the longitudinal 3D facial acquisition of faces over the past decades is just beginning. Further studies on this topic are needed to verify facial asymmetry aging in 3D longitudinal data. Focusing on the features creating facial asymmetry could be helpful for a better understanding of the formation and manifestation of facial aging. This area of research can be beneficial for plastic and aesthetics medicine and for the field of forensic sciences as well.

## Conclusion

The 3D facial shape DA in the adult Czech population was described among 3 age groups in both sexes. Our first hypothesis was that facial DA depends on sex, which was only partially confirmed. Sexually dimorphic difference in DA was found only in the youngest individuals, i.e. between the ages of 20 and 40. Males under 40 have more asymmetric features in the nose, mouth and mandible angle areas. Female facial shape DA was stable during all of the investigated age categories. That is why the second hypothesis (that facial DA differs among age groups) was only partially confirmed in males. Significant differences were found only between the youngest and middle-aged, and the youngest and oldest individuals. In other words, DA did not change after 40 years of life in men.

Despite the significant differences between some groups, both sexes have similar patterns of overall asymmetry, especially in the midline area, where landmarks tended to create a slightly bent “C” shape. After 40 years of age, the sexual dimorphism in facial asymmetry diminished. The third hypothesis of wider DA variability in the oldest individuals, was confirmed. For the oldest subjects, there was the widest range of DA variability, while the youngest subjects showed the opposite. This pattern did not differ much between males and females and could show the cumulative character of morphological changes during life.
