# Correction: The effect of sex and age on facial shape directional asymmetry in adults: A 3D landmarks-based method study

**DOI:** 10.1371/journal.pone.0305196

**Published:** 2024-06-05

**Authors:** Katarína Harnádková, Karolina Kočandrlová, Lenka Kožejová Jaklová, Ján Dupej, Jana Velemínská

An additional affiliation is missing for the second author. Karolina Kočandrlová is also affiliated with Department of Biology and Medical Genetics, 2nd Faculty of Medicine, Charles University and Motol University Hospital, Prague 5, Czech Republic.
